# Vasomotion of mice mesenteric arteries during low oxygen levels

**DOI:** 10.1186/s40001-018-0335-8

**Published:** 2018-08-25

**Authors:** J. Westhoff, K. Weismüller, C. Koch, V. Mann, M. A. Weigand, M. Henrich

**Affiliations:** 1 Department of Anesthesiology and Intensive Care Medicine, Justus-Liebig University Giessen, Rudolph-Buchheimstr. 7, 35392 Giessen, Germany; 2 Department of Anesthesiology and Intensive Care Medicine, St. Vincentius Clinic Karlsruhe, Steinhaeuserstr. 18, 76135 Karlsruhe, Germany; 3 Department of Anesthesiology and Intensive Care Medicine, Heidelberg University Hospital, Im Neuenheimer Feld 110, 69120 Heidelberg, Germany

**Keywords:** Vasomotion, Hypoxia, Mesenteric artery, Mice

## Abstract

**Background:**

Ischemia of intestinal organs is a main cause of complications in surgical intensive care patients. Changes in the tonus of arteries contributing to vascular resistance play an important role in the determination of blood flow and thus oxygen supply of various abdominal organs. It is generally acknowledged that hypoxia itself is able to alter arterial tonus and thus blood flow.

**Methods:**

The present study compared the effects of various degrees of hypoxia on second-order mesenteric arteries from male C57BL/6J mice. After vessel isolation and preparation, we assessed vessel diameter using an arteriograph perfusion chamber. Investigating mechanisms promoting hypoxia-induced vasodilatation, we performed experiments in Ca^2+^-containing and Ca^2+^-free solutions, and furthermore, Ca^2+^-influx was inhibited by NiCl_2_, eNOS^−/−^-, and TASK1^−/−^-mice were investigated too.

**Results:**

Mild hypoxia 14.4% O_2_ induced, in 50% of mesenteric artery segments from wild-type (wt) mice, a vasodilatation; severe hypoxia recruited further segments responding with vasodilatation reaching 80% under anoxia. However, the extension of dilatation of luminal arterial diameter reduced from 1.96% ± 0.55 at 14.4% O_2_ to 0.68% ± 0.13 under anoxia. Arteries exposed to hypoxia in Ca^2+^-free solution responded to lower oxygen levels with increasing degree of vasodilatation (0.85% ± 0.19 at 14.4% O_2_ vs. 1.53% ± 0.42 at 2.7% O_2_). Inhibition of voltage-gated Ca^2+^-influx using NiCl_2_ completely diminished hypoxia-induced vasodilatation. Instead, all arterial segments investigated constricted. Furthermore, we did not observe altered hypoxia-induced vasomotion in eNOS^−/−^- or TASK1^−/−^ mice compared to wt animals.

**Conclusions:**

The present study demonstrated that hypoxic vasodilatation in mice mesenteric arteries is mediated by a NO-independent mechanism. In this experimental setting, we found evidence for Ca^2+^-mediated activation of ion channels causing hypoxic vasodilatation.

**Electronic supplementary material:**

The online version of this article (10.1186/s40001-018-0335-8) contains supplementary material, which is available to authorized users.

## Background

Ischemia of intestinal organs is a main cause of complications in severe ill postoperative intensive care patients. Tissue hypoxia induced by mesenteric ischemia or hypoperfusion causes damage of the intestinal mucosa, consecutive translocation of pathogens, and the release of various inflammatory mediators [1]. This mechanism potentially results in the development of systemic inflammatory response syndrome (SIRS), respectively, sepsis leading to multiple organ failure and increased mortality [2, 3].

Changes in the tonus of arterial vessels determining vascular resistance play an important role in the regulation of blood flow and thus oxygen supply of the various organs. Under physiological conditions, the blood flow of the feeding arteries has to be adjusted precisely to the demand of the receiving organ or tissue [4]. To facilitate the required perfusion, the arterial tonus is controlled by different mechanisms. Perivascular nerve fibres as well as circulating hormones or mediators present external control mechanisms. Metabolic changes surrounding endothelial function and activity of the smooth muscle cells of the arterial wall can directly alter arterial vasomotion and thus display internal control mechanisms [5]. Under pathophysiological conditions like increased oxygen demand, vasomotion has to be regulated very rapidly to prevent the loss of organ function or cell death. Hypoxia is a main condition to change the early organ functions and metabolic state.

It is generally acknowledged that hypoxia itself is able to alter arterial tonus and thus blood flow. The responses observed previously are variable. Increases [6–8] as well as decreases [9, 10] and biphasic effects [11, 12] in arterial tonus are reported.

These different responses are likely to have differing effects on blood flow and oxygen supply, and thus may increase or improve hypoxia. A part of the differing responses to hypoxia can be explained by the different type of vessels studied, but there are also controversial results for the same type of arteries [9, 12]. It is known that some properties of vessels, including agonist sensitivities, ion channels, and endothelial function, differ with the diameter [13–15]. Thus, the vessel diameter may also influence responsiveness to hypoxia. Another reason for controversial results may include differing experimental conditions, particularly in severity of hypoxia or in the method used to evoke hypoxia. Furthermore, it is unknown if the change from normoxia to hypoxia has the same effect on vascular tonus as the switch from hyperoxia to hypoxia. Another experimental way by which hypoxia has been induced is the addition of oxygen scavengers such as sodium dithionite. It has been suggested that chemical means of oxygen removal may have other effects in comparison to reducing oxygen supply [16].

Such differences make it difficult to compare studies, and as a result, it is difficult to get a comprehensive idea of the hypoxic vascular response and the mechanisms involved.

Both endothelial cells (ECs) and vascular smooth muscle cells (VSMCs) play an important role in vasomotion [17, 18]. As vasodilatation is mainly mediated by ECs, vasoconstriction depends more on the VSMCs. Therefore, the vascular reaction of intact arteries to a stimulus is complex as those two cell types can interact with each other. Myoendothelial gap junctions (MEGJs) allow direct cell-to-cell communication between ECs and VSMCs [19].

The present study, therefore, compares the effects of various degrees of hypoxia until anoxia which is chemically achieved in Ca^2+^-containing or Ca^2+^-free solutions on branches of the upper mesenteric artery. To investigate endothelium effects which have been reported in the previous studies, we also performed measurements in eNOS^−/−^- and TASK1^−/−^-mice [9, 20]. Furthermore, the impact of voltage-gated Ca^2+^-influx on hypoxia-induced vasodilatation was examined.

## Methods

### Vessel preparation

Young male wild-type (wt) C57BL/6J mice (30–35 g) and endothelial Nitric oxide synthase knock out mice (eNOS^−/−^, strain B6.129P2 NOS3^tm1Unc^/J) were purchased from Charles Rivers (Sulzbach, Germany). TASK1^−/−^ mice were a kind gift from Prof. Kummer (Institute of Anatomy and Cell Biology, Justus-Liebig-University Giessen, Giessen, Germany). All mice were housed in the animal facility of the Justus-Liebig-University Giessen until processed for experimental use. All procedures involving animals were conducted in compliance to the standards for animal experiments and were approved by the local committee for animal care (AZ: V 54–19 c 20/15 c GI 20/26, 27. May 2008). The mice were sacrificed by cervical dislocation and immediately, afterwards, the abdomen was opened via a median laparotomy. The complete mesenterium with the small intestine was gently mobilised and the mesenteric artery was separated at its origin. The convolute of the small intestine was then carefully spread out in a small petri dish filled with phosphate-buffered saline (PBS). Herein, the complete branching of the mesenteric artery was placed in a radial direction. Now, the second-order arteries (120–150 µm in diameter) of the mesenteric arcade were prepared under sterile conditions still submerged in PBS under a stereomicroscope (Motic SMZ-140-N2GG; Motic Deutschland GmbH, Wetzlar, Germany). Surrounding connective tissue and fat were gently removed, avoiding any direct contact with the arterial wall. After removing of all the connective tissues and the adventitial layer the artery segment 4–6 mm in length was transferred into the recording chamber. Only one vessel of each animal was used.

The isolated mesenteric artery segments were placed and subsequently cannulated in the experimental perfusion chamber of the arteriograph (“single-vessel chamber, CH/1”, Living Systems Instrumentation, Burlington, USA). For cannulation, one end of the arterial segment was fixed using a No. 5 forceps and mounted onto the tip of a glass microcannula (Glass Cannula, tip diameter 100–125; Firma Living Systems Instrumentation, Burlington, USA). The artery was fixed with two ligatures, and then, a pressure of 5–10 mmHg was applied using PBS to clean the vessel. Finally, the other end of the arterial segment was cannulated and fixed by a double ligature. For the ligatures, single filaments of surgical threads were used, these assured that the artery was kept in constant position throughout the experimental period and the parts of the artery that had been touched by the forceps were separated from those included into the study.

### Experimental setup

The chamber of the arteriograph was mounted on the stage of an inverting microscope (Axiovert 35, Carl Zeiss, Oberkochen, Germany). This setup allowed to superfuse and measure changes in diameter of arteries at constant transmural pressures. The isolated arterial segments can be superfused by solutions of various gas concentrations or various compositions. All experiments were performed at a temperature of 37 °C; the solutions were preheated in a water bath and a thermistor probe allowing intermittent temperature control at different positions. Continuous exchange of the preheated experimental buffer solution was achieved through a gas proof rubber tubing system (Nalgene 50 Platinum-Cored Silicone Tubing, Fisher Scientific GmbH, Schwerte, Germany; Tygon R3603, Novodirect GmbH, Kehl, Germany) connected to a Masterflex pump (EasyLoad II, Cole-Parmer Instrument, Chicago, IL., USA) and a heat exchanger with permanent temperature control (Haake D8-L, Haake Mess-Technik, Karlsruhe, Germany). The luminal diameter of the arterial segment was continuously measured with a video camera (TC 7014 X, Burle Industries Inc., Lancaster, USA) attached to the inverting microscope and connected with a dimension analysing system (TC 1112 X, Living Systems Instrumentation, Burlington, USA); for technical details, see [21, 22]. Vessel diameter and pressure values were continuously recorded by an analogue servocorder (SR 6211, Graphtec, Tokyo, Japan).

### Drugs and solutions with predetermined oxygen concentrations

Bicarbonate-buffered Tyrode solution consisted of (in mM) NaCl 117, KCl 4.5, CaCl_2_ 2.5, NaHCO_3_ 23, MgCl_2_ 1, and glucose 11, equilibrated with 21% O_2_ and 5% CO_2_, balanced with N_2_ attaining a pH 7.4 at 37 °C was used as normoxic control solution. Ca^2+^-free solutions were prepared by omitting CaCl_2_ and addition of ethylene glycol-bis(*β*-aminoethyl ether)-*N*,*N*,*N*′,*N*′-tetraacetic acid] (EGTA, 1 mM). In several experimental series, NiCl_2_ was applied to the Ca^2+^-containing Tyrode solution in a concentration of 2.5 mM to inhibit voltage-gated Ca^2+^-entry. All experimental solutions were prepared directly before the experiments. Hypoxic gas concentrations were adjusted by equilibrating the solutions from gas cylinders containing customized oxygen mixtures; hypoxic conditions were attained with decreasing oxygen concentrations: 5% O_2_ balanced N_2_ and 5% CO_2_, or 0% O_2_ balanced with N_2_, 5% CO_2_. An oxygenator (162 fibres, 115 cm^2^ gas exchange surface area, Miniature gas exchange oxygenator, Living Systems Instrumentation, Burlington, USA) was placed directly in front of the inflow tube of the experimental chamber to maintain constant low oxygen levels. Very low oxygen levels and anoxic gas concentrations were adjusted by equilibrating the solutions with zero O_2_ balanced with N_2_ and 5% CO_2_. Anoxia in the bath solution was achieved by application of 0.5 mM Na_2_S_2_O_4_ 3 min before administration (see Additional file 1). Oxygen concentrations were adjusted by preequilibrating the solutions for at least 30 min using the above-mentioned gas mixtures. Oxygen tensions in the bath solution were recorded using a 50 μm fibre optic oxygen sensor (Microx TX2, PreSens, Regensburg, Germany) response times (T_90_) for the optodes of approximately 5 s. According to the manufacturer’s recommendations, the sensor was subjected to a two-point calibration at 37 °C. The measured PO_2_ values were expressed in Torr (mmHg) using an air-equilibrated solution for 150 Torr PO_2_ and fresh anoxic solution (with 0.5 mM Na_2_S_2_O_4_) for 0 Torr PO_2_. Stable hypoxic gas concentrations in the bath solution were achieved within 50–120 s by solution exchange rates of 20 ml/min. PO_2_ in the experimental chamber was typically reduced to between 1.3 and 2 Torr when using Na_2_S_2_O_4_ (0.5 mM) showing a small difference between anoxic solutions in the bath and the zero-point calibration (see Additional file 1). Quick exchange of solutions with different oxygen tension was achieved by mechanically driven taps directly at the inflow of the chamber, allowing a fast exposure to certain oxygen levels or to pharmacological agents.

### Experimental protocol

After cannulation, the arteries were equilibrated for 30 min at 50 mmHg, pH 7.4, 37 °C. Following this resting period, the arteries were preconstricted with the EC_50_ of phenylephrine (10^−6^ M, Phe) to evoke its half maximal vasoconstriction (see Additional file 2). Phe (10^−6^ M) was continuously present during the subsequent experimental procedure, while the perfusion pressure was held constant at 50 mmHg. The exposure to solutions with low oxygen tension persisted for 10–30 min to achieve complete equilibration, and then, the solution was changed back to normoxia or to an even lower oxygen concentration. Pharmacological agents were administered 10 min prior to change in oxygen concentration. In all experiments, oxygen concentration was switched back to normoxia; finally, 50 mM KCl was applied as proof of vitality.

### Data analysis

Vasomotion was expressed as relative percentage either as vasoconstriction or as vasodilatation. Vasomotion in either direction was calculated in relation to the half maximal vasoconstriction evoked by Phe_50_ defined as resting tone. Vm (%) = [(*d*_PheEC50_ − *d*_vm_)/d_PheEC50_] × 100 (*d*_PheEC50_: diameter at Phe_EC50_, *d*_vm_: diameter under low oxygen tension). Vasoconstriction was calculated in relation to maximal constriction evoked by Phe using the following formula: vc (%) = [(*d*_Phemax_ − *d*_VChyp_)/*d*_Phemax_] × 100. *d*_Phemax_: diameter in Phe causing a maximal constriction; *d*_VChyp_: diameter under low oxygen tension. The degree in vasodilatation, starting from Phe_50_ precontracted arteries was calculated in relation to the maximal dilatation evoked by sodium nitroprusside (Nipruss, 10^−4^ M, see Additional file 2) using the following formula: vd (%) = [(*d*_VDhyp_ − *d*_PheEC50_)/(*d*_Nip_ − *d*_PheEC50_)] × 100 (*d*_VDhyp_: vasodilatated diameter under hypoxia, *d*_PheEC50_: diameter at Phe_EC50_, and *d*_Nip_: maximal diameter under Nipruss).

### Dose–response curves

From each animal, only one artery was included into the data evaluation. The data were normalised to the maximum observed change in diameter either as vasoconstriction evoked by Phe or as vasodilatation evoked by Nipruss to generate dose–response curves. This allowed us to express intermediate response as a fraction of the maximum response seen under Phe or Nipruss. Finally, the curves were transformed using the Hill equation to generate sigmoid curves and to estimate effective concentrations for a defined observed arterial response (concentration evoking the half maximal response was defined as EC_50_, see Additional file 2).

### Statistics

In the present study, all data are presented as mean ± standard error of the mean (SEM). From each animal, only one artery was taken and included into the statistical evaluation, contributing to the number (*n*) depicted in the text and figures. Measurements of diameters under different oxygen tensions were compared to the diameter under normoxia in addition of Phe_50_. Diameters were regarded as significantly different when *p* < 0.05, using either Student’s *t* test for gaussian distributed data or Mann–Whitney *U* test for non-parametric distributed data.

## Results

### Direction of arterial vasomotion depends on oxygen concentration

Sections of the second-order mesenteric arteries, which were kept under normoxia, responded in a dose-dependent manner to the application of Phe (10^−8^–10^−3^ M). Phe achieved the maximum constriction at 10^−3^ M reducing the inner vessel diameter by 9.67%; the EC_50_ was determined at 10^−6^ M which was used in all subsequent experiments investigating the effects of low oxygen levels (see Additional file 1). Exposure of precontracted arteries (10^−6^ M Phe) to Nipruss (10^−9^–10^−4^ M) also showed a dose-dependent vasodilatation reaching its maximum at 10^−4^ M dilating the inner diameter by 3.74%. Changes in solution with the same content and oxygen tension did not influence arterial diameter or pressure. Vitality test performed at the end of each experiment in normoxic solutions was performed by 50 mM KCl, provoking vasoconstriction.

### Arterial diameter in hypoxic solutions

**Fig. 1:**
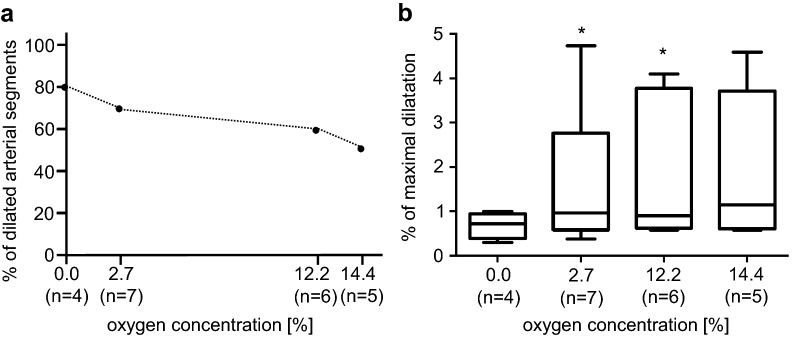
Percentage of dilating arterial segments and extension of vasodilation. **a** Percentage of dilating arterial segments compared to all the examined arteries. More arterial segments respond with vasodilatation when hypoxia becomes more severe. Under anoxia, 80% of all investigated arteries dilated. The dotted line may stress that the frequency of occurrence of dilatation was dependent from hypoxic oxygen concentrations. **b** Percentage changes of arterial vessel diameter under different hypoxic conditions in relation to the maximum vasodilatation using 0.1 mM nitroprusside sodium. *n* = number of arteries that responded to hypoxia with vasodilatation. Statistical testing was performed using the maximal evoked diameter using nitroprusside sodium as control. Student paired *t* test, Wilcoxon rank-sum test for non-parametric data (*p < 0.05)

Exposing second-order arterial segments of wt-mice superior mesenteric artery to hypoxia resulted in gradually altered vasomotion leading to more vessels dilating under lower oxygen levels. Mild hypoxia 14.4% O_2_ evoked in approximately 50% of the investigated vessels vasodilatation, whereas all other arteries responded with vasoconstriction. Further lowering the oxygen content in the bath solution via 12.2% and 2.7% to anoxia evoked in more vessels’ vasodilatation instead of vasoconstriction. Under anoxic conditions, 80% of the investigated arterial segments showed a vasodilatation (Fig. 1a). The inner arterial diameter dilated by 1.96% ± 0.55 at 14.4% O_2_ (*n* = 5, *p* = 0.065), 1.8% ± 0.51 at 12.2% (*n* = 6, *p* < 0.05), and 1.82% ± 0.5 at 2.7% (*n* = 7, *p* < 0.05), and under anoxia, the dilatation of the inner diameter was 0.68% ± 0.13 (*n* = 4, *p* = 0.13). When comparing the hypoxia evoked dilatation to the maximum achievable diameter evoked by Nipruss (10^−4^ M), hypoxia led at 14.4% O_2_ to 55% ± 15 (*n* = 5, *p* < 0.05), at 12.2% O_2_ to 50% ± 14 (*n* = 6, *p* < 0.05), at 2.7% to 51% ± 14 (*n* = 7, *p* < 0.05), and under anoxia to 19% ± 4 (*n* = 4, *p* = 0.32) of the inner diameter (Fig. 1b).

### Arterial hypoxic vasomotion in Ca^2+^-free medium

**Fig. 2:**
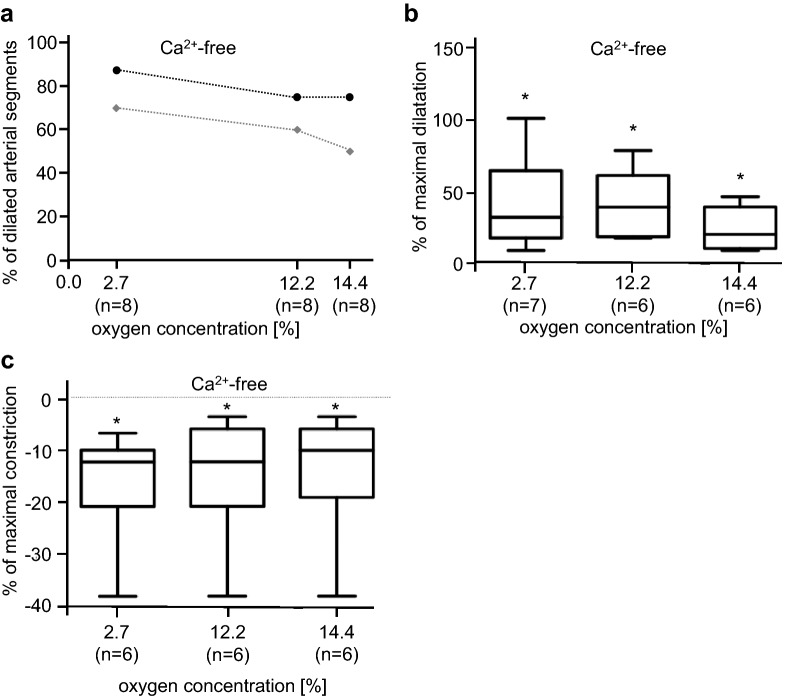
Vasomotion of arterial segments in Ca^2+^-free solution. **a** Percentage of dilating arterial segments in Ca^2+^-free buffer solutions. Under each oxygen concentration, we investigated eight arterial segments each from different animals. In Ca^2+^-free solution, more vessel segments responded with vasodilation compared to Ca^2+^-containing solutions. When reducing oxygen concentrations more arteries responded with vasodilatation. The black line represents the experiments in Ca^2+^-free solution and the grey line represents the controls in Ca^2+^-containing buffer. Controls (*n* = number of arteries responding with vasodilatation from a total number of eight). **b** Percentage change of luminal diameter in Ca^2+^-free buffer solution is represented in relation to the maximal dilatation evoked by 0.1 mM nitroprusside sodium. *n* = number of arterial segments that responded with hypoxia-induced vasodilatation. **c** Percentage change of luminal diameter is represented in relation to the maximal contraction induced by 1 mM Phe. Negative algebraic sign on the *Y*-axis: constriction of the arterial segment (*n* = number of arteries from different individual animals; *p < 0.05; Student *t* test for paired samples)

To evaluate the role of extracellular Ca^2+^-concentration ([Ca^2+^]_e_) onto the hypoxic response of mesenteric arteries, we performed a series of experiments in Ca^2+^-free bicarbonate-buffered solutions. At 14.4% O_2_-concentration from all arterial segments investigated, 75% dilated by 0.85% ± 0.19 compared to diameter of normoxic controls. This corresponds to 24 ± 5.3% of the maximal dilatation evoked by Nipruss (10^−4^ M). In contrast to physiological Ca^2+^-levels, arteries in zero [Ca^2+^]_e_ responded to falling oxygen levels with an increasing degree of vasodilatation, which was less pronounced as seen in Ca^2+^-containing bath solutions. At 12.2% O_2_, arteries dilated in zero [Ca^2+^]_e_ by 1.5% ± 0.3 (*n* = 6) compared to the initial inner diameter; at 2.7% O_2_, the vessel sections dilated by 1.53 ± 0.42 (*n* = 7) of the initial diameter (Fig. 2a). Compared to the maximal dilatation evoked by Nipruss (10^−4^ M), oxygen levels of 14.4% elicited a vasodilatation of 23% ± 5 (*n* = 6), at 12.2% O_2_ arteries dilated by 41% ± 8 (*n* = 6) of the maximal dilatation, and at 2.7% O_2_ arterial segments dilated by 43% ± 12 (*n* = 7) of maximal dilatation (Fig. 2b).

### Hypoxic vasomotion under inhibition of voltage-gated Ca^2+^-influx

Exposure of arterial segments to hypoxia in Tyrode solution with physiological [Ca^2+^]_e_ supplemented with 2.5 mM NiCl_2_ completely diminished hypoxia-induced vasodilatation; instead, all arteries constricted. Under 14.4% O_2_, the inner diameter constricted by 1.31% ± 0.49 (*n* = 6, *p* < 0.05), at 12.2% O_2_ by 1.42% ± 0.48 (*n* = 6, *p* < 0.05), and at 2.7% O_2_ 1.54% ± 0.44 (*n* = 6, *p* < 0.05) all compared to vasomotion under hypoxia in physiological [Ca^2+^]_e_. Comparing these data to the maximal constriction achieved by Phe (10^−3^ M), mild hypoxia (14.4% O_2_) led to 14.4% ± 5 (*n* = 6, *p* < 0.05), 12.2% O_2_ to 15% ± 5 (*n* = 6, *p* < 0.05), and 2.7% to 16% ± 4 (*n* = 6, *p* < 0.05) (Fig. 2c).

### Hypoxic arterial vasomotion in eNOS^−/−^-mice

Arterial sections which investigated from eNOS^−/−^-mice also responded to hypoxic solutions either with dilatation or constriction.

**Fig. 3:**
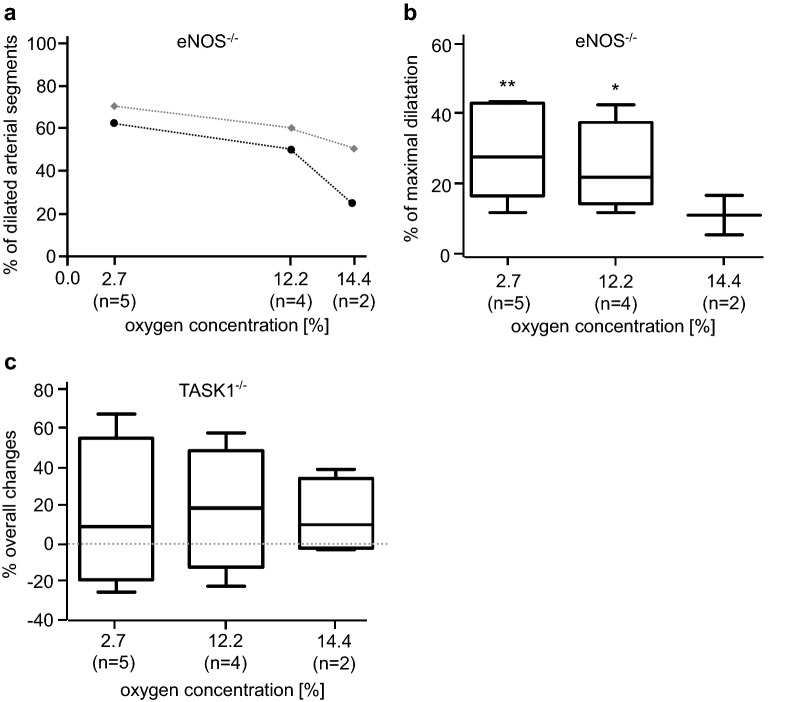
Hypoxia-induced vasomotion of arterial segments from eNOS^−/−^- and TASK1^−/−^-mice. **a** Percentage of dilating arterial segments of eNOS^−/−^-mice in Tyrode’s solution containing Ca^2+^ (*Y*-axis) as function of the oxygen concentration (*X*-axis). The black line represents the experiments with eNOS^−/−^-mice and the grey line represents the controls from wt-mice (*n* = number of arteries responding with vasodilatation from a total number of eight). **b** Percentage change in luminal diameter from eNOS^−/−^-mice is represented in relation to the maximal dilatation induced by 0.1 mM nitroprusside sodium (*n* = arterial segments responding with vasodilation from a total number of eight). **c** Percentage change in luminal diameter from TASK1^−/−^-mice in relation to the maximal reaction (dilatation or contraction). Negative algebraic sign on the *Y*-axis: constriction of the arterial segment; *p < 0.05; **p < 0.01 (*n* = number of independent experiments; Student *t* test for paired samples)

Vasodilatation under hypoxia: the number of vessels responding to hypoxia with vasodilatation was smaller than in wt animals (Fig. 3a). Under mild hypoxia (14.4% O_2_), only 25% of arteries dilated by 0.4% ± 0.1 compared to the initial diameter; these vessels reached 11% ± 3 (*n* = 2) of the maximal dilatation evoked by Nipruss (10^−4^ M, Fig. 3b). At 12.2% O_2_, the number of dilatating arteries increased to 50% of investigated vessel segments and this dilatation of the inner diameter was more pronounced by 0.88% ± 0.16 (*n* = 4, *p* < 0.05) compared to controls and reached 25% ± 5 of the maximal dilatation (*n* = 4, *p* < 0.01). Furthermore, at 2.7% O_2_, the percentage of dilatating vessels increased to 63%; the arteries dilated by 1.05% ± 0.17 (*n* = 5, *p* < 0.05) of the initial diameter and reached 29% ± 5% (*n* = 5, *p* < 0.05) of the maximal dilatation (Fig. 3b).

Vasoconstriction under hypoxia: In eNOS^−/−^-mice too, one group of mesenteric arteries constricted under hypoxia. Under 14.4% O_2_, these vessels constricted by 1.37% ± 0.22 and the constriction gradually increased at lower oxygen levels. At 12.2% O_2_, the vessel diameter was reduced by 2.05% ± 0.52 (*n* = 3) and at 2.7% O_2_ by 2.32% ± 0.46 (*n* = 3) compared to the initial diameter. Comparing the hypoxia-induced vasoconstriction to the maximal constriction evoked by Phe (10^−3^ M) in eNOS^−/−^-mice, the mesenteric arteries achieved 11% ± 2 (*n* = 3) at 14.4% O_2_, increased to 24% ± 4 (*n* = 3) at 12.2% O_2_, and further to 24% ± 5 (*n* = 3) at 2.7% O_2_.

### Hypoxia-induced vasomotion in TASK1^−/−^-mice

Under hypoxia (14.4% O_2_), only one of four arteries from TASK1^−/−^-mice constricted, decreasing the inner diameter by 0.31%. When reducing the oxygen tension to 12.2% or 2.7%, still only one vessel constricted by 2.1% or 2.5%, respectively. Comparing the induced constriction with the maximum constriction achievable by Phe (10^−3^ M), 14.4% O_2_ provoked 3.2%, 12.2% O_2_ 23%, and oxygen concentration of 2.7% induced a vasoconstriction of 25% of the maximum. Three of four vessels investigated responded to hypoxia with a vasodilatation that increased the inner diameter by 1% ± 0.2 (*n* = 3) at 14.4% O_2_, at 12.2% O_2_ the inner diameter increased by 1.2% ± 0.4 (*n* = 3), and at low oxygen concentration of 2.7% the inner diameter increased by 1.6% ± 0.5 (*n* = 3). At 14.4% O_2_, these changes were able to achieve 30% ± 7 (*n* = 3) of the maximal dilatation evoked by Nipruss (10^−4^ M); at 12.2% O_2_, the dilatation was further pronounced by 32% ± 12 (*n* = 3); at very low oxygen concentration of 2.7%, it led to 43% ± 18 (*n* = 3) of the maximal vasodilatation (Fig. 3c).

### Overall change of baseline diameter during low oxygen tension

**Fig. 4:**
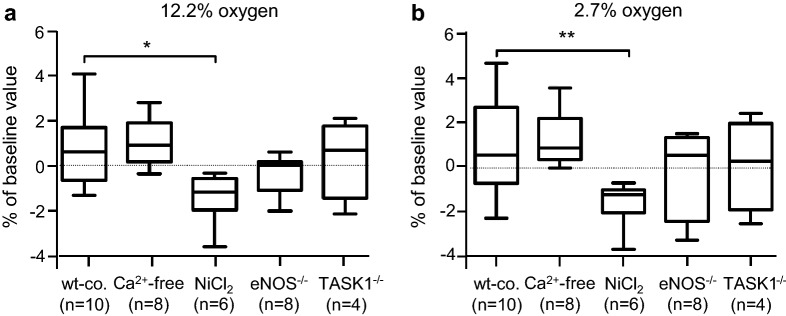
Comparison of vasomotion at moderate and severe hypoxic conditions. The results are shown as percentage changes in relation to the initial diameter under normoxic conditions. The *X*-axis represents the investigated groups, wt-co.: wild-type controls, Ca^2+^-free: Ca^2+^-free Tyrode’s solution, NiCl_2_: Tyrode’s solution containing 2.5 mM NiCl_2_. **a** Vasomotion under 12.2% oxygen; **b** vasomotion under 2.7% oxygen; negative algebraic sign on the *Y*-axis: constriction of the arterial segment; (*p < 0.05; **p < 0.01; Mann–Whitney *U* test for non-parametric data)

Regarding the overall percentage alterations from baseline of arterial diameter under hypoxia (2.7% O_2_ and 12.2% O_2_) of each experimental group, we assessed significant changes between wt-mice in Tyrode’s solution and Ca^2+^-free solution compared to NiCl_2_-treated mice (Fig. 4a, b, Table 1). In contrast, eNOS^−/−^-mice and TASK1^−/−^-mice did not show differences compared to wt-mice (Fig. 4a, b).

**Table 1:** Vasomotion of isolated segments of the mesenteric artery

	Oxygen concentration (%)			
	14.4 *n* = 24	12.2 *n* = 26	2.7	0
Percentage of the initial diameter				
Overall	0.58 ± 0.59 (*n* = 10)	0.77 ± 0.57 (*n* = 10)	0.92 ± 0.64 (*n* = 10)	0.43 ± 0.28 (*n* = 5)
Constriction	− 0.79 ± 0.05 (*n* = 5)	− 0.765 ± 0.14 (*n* = 4)	− 1.213 ± 0.28 (*n* = 3)	− 0.6 (*n* = 1)
Dilation	1.96 ± 0.55 (*n* = 5)	1.8 ± 0.52 (*n* = 6)	1.83 ± 0.52 (*n* = 7)	0.68 ± 0.13 (*n* = 4)
Percentage of maximal dilatation and constriction				
Overall	23.2 ± 14.62 (*n* = 10)	26.92 ± 14.35 (*n* = 10)	31.94 ± 15.32 (*n* = 10)	14 ± 5.96 (*n* = 5)
Constriction	− 8.21 ± 0.56 (*n* = 5)	− 7.92 ± 4.42 (*n* = 5)	− 12.54 ± 2.92 (*n* = 5)	− 6.21 (*n* = 1)
Dilation	54.6 ± 15.31 (*n* = 5)	50.14 ± 14.41 (*n* = 6)	51.01 ± 14.42 (*n* = 7)	19.05 ± 3.61 (*n* = 4)
Percentage of the initial diameter in Ca^2+^-free Tyrode’s solution				
Overall	0.36 ± 0.39 (*n* = 8)	1.07 ± 0.38 (*n* = 8)	1.52 ± 0.43 (*n* = 8)	−
Constriction	− 1.14 ± 0.4 (*n* = 2)	− 0.39 (*n* = 1)	–	−
Dilation	0.85 ± 0.19 (*n* = 6)	1.49 ± 0.3 (*n* = 6)	1.53 ± 0.42 (*n* = 7)	−
Percentage of maximal dilatation and constriction in Ca^2+^-free Tyrode’s solution				
Overall	14.93 ± 7.5 (*n* = 8)	38.58 ± 10.03 (*n* = 8)	37.36 ± 12.06 (*n* = 8)	–
Constriction	− 11.8 ± 4.14 (*n* = 2)	− 4.01 (*n* = 1)	–	–
Dilation	23.82 ± 5.29 (*n* = 6)	41.51 ± 8.35 (*n* = 6)	42.7 ± 11.68 (*n* = 7)	–
Percentage of the initial diameter in Tyrode’s solution containing NiCl_2_				
Overall	− 1.31 ± 0.49 (*n* = 6)	− 1.42 ± 0.48 (*n* = 6)	− 1.54 ± 0.44 (*n* = 6)	–
Percentage of maximal constriction in Tyrode’s solution containing NiCl_2_				
Overall	− 13.6 ± 5.03 (*n* = 6)	− 14.7 ± 4.93 (*n* = 6)	− 15.93 ± 4.52 (*n* = 6)	-
Percentage of the initial diameter in eNOS^− /−^-mice				
Overall	− 0.42 ± 0.31 (*n* = 8)	− 0.33 ± 0.6 (*n* = 8)	− 0.21 ± 0.68 (*n* = 8)	–
Constriction	− 1.37 ± 0.22 (*n* = 3)	− 2.05 ± 0.52 (*n* = 3)	− 2.32 ± 0.46 (*n* = 3)	–
Dilation	0.4 ± 0.1 (*n* = 2)	0.88 ± 0.16 (*n* = 4)	1.05 ± 0.17 (*n* = 5)	–
Percentage of maximal dilatation and constriction in eNOS^−/−^-mice				
Overall	− 2.56 ± 4.1 (*n* = 8)	3.43 ± 9.21 (*n* = 8)	9.38 ± 10.73 (*n* = 8)	–
Constriction	− 10.64 ± 2.3 (*n* = 3)	− 23.64 ± 4 (*n* = 3)	− 24 ± 4.73 (*n* = 3)	–
Dilation	11.05 ± 2.3 (*n* = 2)	24.59 ± 4.58 (*n* = 4)	29.4 ± 4.85 (*n* = 5)	–
Percentage of the initial diameter in TASK1^−/−^-mice				
Overall	0.46 ± 0.38 (*n* = 4)	0.32 ± 0.9 (*n* = 4)	0.15 ± 1.03 (*n* = 4)	–
Constriction	− 0.31 (*n* = 1)	− 2.19 (*n* = 1)	− 2.5 (*n* = 1)	–
Dilation	1.07 ± 0.24 (*n* = 2)	1.16 ± 0.42 (*n* = 3)	1.55 ± 0.65 (*n* = 2)	–
Percentage of maximal dilatation and constriction in TASK1^−/−^-mice				
Overall	14.08 ± 9.89 (*n* = 4)	18.56 ± 16.67 (*n* = 4)	15.16 ± 19 (*n* = 4)	–
Constriction	− 3.23 (*n* = 1)	− 22.6 (*n* = 1)	− 25.86 (*n* = 1)	–
Dilation	29.78 ± 6.75 (*n* = 2)	32.3 ± 11.57 (*n* = 3)	43.24 ± 18.08 (*n* = 2)	–

Measured values shown as percentage changes of the arterial diameter. Data are shown as mean ± standard error of the mean (SEM)

### Oscillation of arterial sections during low oxygen tension

Under normoxic conditions, sections of mesenteric arteries oscillated while preconstricted with Phe with a frequency of 0.025 Hz; the inner diameter changed under these oscillations to both vasoconstriction and vasodilatation. However, with increasing concentration of Phe, the oscillations mainly dilated the vessels.

In all arterial sections investigated, which were preconstricted with Phe (10^−6^ M), we observed oscillations of the inner arterial diameter. The frequency of these oscillations ranged between 0.02 Hz towards vasoconstriction and 0.032 Hz towards vasodilatation. Exposing the arteries to low oxygen concentration did not change the frequency of the oscillations; however, it changed the degree of vasomotion. Oscillation amplitudes during normoxia changed the luminal diameter by 1.4%. These amplitudes showed a trend to be increased by hypoxia. The diameter changed at 14.4% O_2_ by 1.5%, at 12.2% O_2_ by 1.8%, and at 2.7 by 1.7% compared to resting diameter (*n* = 10 for all conditions). Amplitudes of the oscillations increased in Ca^2+^-free solutions to 3.1% of the inner diameter at 21% O_2_. When we decreased oxygen concentration to 14.4%, the oscillation amplitudes were reduced to 2.8%; further reducing O_2_ to 12.2% or 2.7% slightly reduced the amplitudes to 2.7% of the resting diameter. From exposure of arteries to NiCl_2_ (2.5 mM) under normoxia, the oscillation amplitudes increased to 2% of the resting diameter. During hypoxia, these amplitudes showed only a trend to be reduced by NiCl_2_. Under normoxic conditions in eNOS^−/−^-mice, the oscillation amplitudes reached 0.8% and increased gradually with decreasing O_2_-concentrations.

## Discussion

In the present study, we investigated isolated second-order mesenteric artery branches from male mice, which were cannulated and exposed to low oxygen levels. Therefore, we demonstrated that these vessels only respond little to pharmacological or pathophysiological stimuli as it has been aforementioned. We chose mice arteries to further investigate knock out animals that made it feasible to focus on certain signalling pathways. We were able to demonstrate that hypoxic vasodilatation in mice mesenteric arteries is mediated by a NO-independent mechanism. In our experimental setting, we identified Ca^2+^-mediated hypoxic vasodilation depending on Ca^2+^-influx. Recent studies reported that vasomotor responses differ between various vascular territories of C57BL/6J mice [23]. In contrast to our approach, the first-order mesenteric artery sections seem to have a more distinctive vasomotion compared to the vessels investigated here and elsewhere [24, 25]. The main challenge in our experimental setup was to lower oxygen levels in the bath solutions and to maintain these hypoxic conditions throughout the experimental period, especially very low oxygen levels below 2% O_2_ or anoxic conditions where only stable for brief periods. Thus, these experiments were necessary to simulate ischemia, without any oxygen supply.

Vasomotion under mild hypoxia was responded in different directions; one group of arteries constricted, whereas the second group showed vasodilatation. Further decreasing oxygen levels led to dilatation of more vessel sections; hence, the amplitude of dilation was less pronounced. These findings are in contrast to other vascular beds in which hypoxia evoked vasoconstriction to avoid desaturation of the blood, e.g., pulmonary arteries [26, 27]. Otter et al. also found that severe hypoxia relaxes isolated third-order rat mesenteric arteries by a mechanism that is independent of the endothelial factors NO and prostaglandins [28]. The authors further demonstrated that the measured effects were neither caused by a mechanism that was independent of pH_i_ and suggested that one possible mechanism might be an effect of lactate on the cAMP system. These findings are consistent with studies that revealed pH-independent effects of lactate on Ca^2+^-affinity and Ca^2+^-activated ion channels [28–30]. Regarding these results, we performed the measurements of arterial hypoxic vasomotion in Ca^2+^-free solution. According to the demonstrated mechanisms, we revealed an increased rate of dilated vessels in Ca^2+^-free medium under hypoxia compared to Ca^2+^-perfused control. Even studies in porcine coronary arteries demonstrated that, under hypoxic conditions, lactate induced vasodilatation by stimulation of Ca^2+^-activated K^+^-channels [30]. Interestingly, inhibition of ATP-dependent K^+^-channels did not reverse the effect of lactate on vascular dilatation. The previous studies of rat small mesenteric arteries also described a mechanism of vasodilatation by adenosine that involves the regulation of store-operated Ca^2+^-entry through the cAMP signalling pathway due to the activation of adenosine A (2A) receptors [31].

Regarding these effects, we further evaluated the hypoxic vasomotion under inhibition of voltage-gated Ca^2+^-influx by the application of NiCl_2_. As a result, we found a complete diminishing of hypoxia-induced vasodilatation; instead, all arteries investigated constricted. Beside these mechanisms, we further evaluated the influence of endothelial-derived NO on hypoxic arterial vasomotion, using eNOS^−/−^-mice. Compared to wt-mice, arteries from eNOS^−/−^-mice responded with vasodilatation in Ca^2+^-containing solution under hypoxia; however, the overall percentage of dilated vessels of the entire group remained lower. These results confirm the reports from the previous studies that found no association between endothelial-derived NO and hypoxic vasodilatation [28, 32].

Further investigating potential influences of oxygen sensing on hypoxic vasodilatation, we chose TASK1^−/−^-mice to exclude direct hypoxic effects on TASK-1 channels. The TASK-1 is a pH- and oxygen-sensitive potassium channel that has been described in multiple tissues, e.g., cardiomyocytes, brain, carotid body, or mesenteric arteries [33]. However, exposing mesenteric arteries from TASK1^−/−^-mice to hypoxia did not alter vasomotion when compared to wt animals. These results confirm our hypothesis claiming a Ca^2+^-mediated activation of ion channels to cause hypoxic vasodilatation.

In addition, we focused on oscillations of the arterial section diameter during low oxygen tension. During our experiments, we assessed that, under conditions of decreased oxygen concentrations, the oscillation frequency showed only a trend to be increased. According to the previous findings, we assume that lowering of oxygen concentration causes decreased intracellular ATP concentration which reduces sarco(endo)plasmic reticulum ATPase-activity leading to cytosolic Ca^2+^-oscillations and eventually to oscillations of the vessel diameter as it has previously been shown in rat mesenteric arteries [34, 35].

In summary, we demonstrated the impact of hypoxia on vasodilatation in mice’ mesenteric arteries caused by Ca^2+^-mediated activation of ion channels. Regarding the limitations of our study, we concede that our findings only reflect the situation in mice second-order mesenteric arteries. Because of the different behaviour of vessels, vessel sections, or species, these results cannot be generalized. Indeed, these mechanisms of endothelial-derived and endothelial-independent energy supply need further evaluation, but we speculate that our results provide crucial information for the understanding of hypoxia-induced vasodilatation.

## Conclusions

The present study revealed that hypoxic vasodilatation in mice mesenteric arteries is mainly mediated by a NO-independent mechanism. We found evidence that Ca^2+^-mediated activation of ion channels caused hypoxic vasodilation in our experimental setting.

## Additional files

**Additional file 1.** Time course of oxygen partial pressures in the bath solutions at 37 °C. Low oxygen levels were adjusted using calibration with hypoxic gas mixtures. Oxygen measurements were conducted using an optical oxygen microsensor (Microx TX2, PreSens, Regensburg, Germany) placed adjacent to the arterial segments. A: Different oxygen concentrations were adjusted, I: normoxia 21%, 150 Torr; II: 14.4%, 110 Torr; III: 12.2%, 93 Torr. IV: deep hypoxic oxygen partial pressure of 21 Torr was reached with gas mixture of 2.7% oxygen, balanced with N_2_ and 5% CO_2_. At the end of this exposure period, the gas mixture was stepwise changed back to higher oxygen levels. B: Anoxic conditions in the bath solution were achieved after successive reduction of oxygen concentration and by application of 0.5 mM Na_2_S_2_O_4_. I: 0.0%, 0 Torr. Returning to normoxia was carried out stepwise at the end of the anoxic exposure period.

**Additional file 2.** Dose–response curves for the estimation of maximal vasoconstriction and vasodilatation of mice superior mesenteric artery at 37 °C. A: phenylephrine was used to evoke vasoconstriction with EC50 of 10^−6^ M. After vessel calibration, the maximal change in luminal diameter evoked by Phe was 9.67%. B: sodium nitroprusside was used to demonstrate the vasodilatation of preconstricted arterial segments; maximal vasodilatation of 3.74% was evoked by 10^−4^ M sodium nitroprusside compared to the initial diameter. The maximum constriction/vasodilatation was arbitrarily set as 100%.
