# Supplementary material for: Vasomotion of mice mesenteric arteries during low oxygen levels
Source: Eur J Med Res. 2018 Aug 25;23:38. doi: 10.1186/s40001-018-0335-8 (PMC6109325; doi:10.1186/s40001-018-0335-8)
Supplement: Supplementary file 1 — Additional file 1. Time course of oxygen partial pressures in the bath solutions at 37 °C. Low oxygen levels were adjusted using calibration with hypoxic gas mixtures. Oxygen measurements were conducted using an optical oxygen microsensor (Microx TX2, PreSens, Regensburg, Germany) placed adjacent to the arterial segments. A: Different oxygen concentrations were adjusted, I: normoxia 21%, 150 Torr; II: 14.4%, 110 Torr; III: 12.2%, 93 Torr. IV: deep hypoxic oxygen partial pressure of 21 Torr was reached with gas mixture of 2.7% oxygen, balanced with N2 and 5% CO2. At the end of this exposure period, the gas mixture was stepwise changed back to higher oxygen levels. B: Anoxic conditions in the bath solution were achieved after successive reduction of oxygen concentration and by application of 0.5 mM Na2S2O4. I: 0.0%, 0 Torr. Returning to normoxia was carried out stepwise at the end of the anoxic exposure period. [file 40001_2018_335_MOESM1_ESM.pdf]

Additional file 1

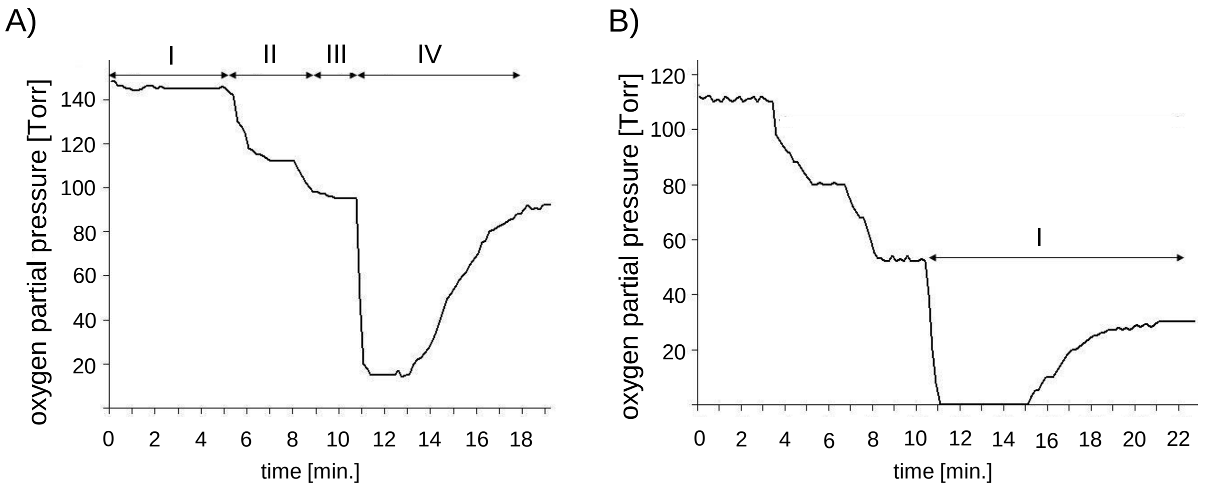

Time course of oxygen partial pressures in the bath solutions at 37°C. Low oxygen levels were adjusted using calibration with hypoxic gas mixtures. Oxygen measurements were conducted using an optical oxygen microsensor (Microx TX2, PreSens, Regensburg, Germany) placed adjacent to the arterial segments. **A:** Different oxygen concentrations were adjusted, I: normoxia 21%, 150 Torr; II: 14.4%, 110 Torr; III: 12.2 %, 93 Torr. IV: deep hypoxic oxygen partial pressure of 21 Torr was reached with gas mixture of 2.7% oxygen, balanced with N_2 and 5% CO_2. At the end of this exposure period the gas mixture was stepwise changed back to higher oxygen levels.

B: Anoxic conditions in the bath solution were achieved after successive reduction of oxygen concentration and by application of 0.5 mM $Na_2S_2O_4$. I: 0.0%, 0 Torr. Returning to normoxia was carried out stepwise at the end of the anoxic exposure period.
