# Supplementary material for: Vasomotion of mice mesenteric arteries during low oxygen levels
Source: Eur J Med Res. 2018 Aug 25;23:38. doi: 10.1186/s40001-018-0335-8 (PMC6109325; doi:10.1186/s40001-018-0335-8)
Supplement: Supplementary file 2 — Additional file 2. Dose–response curves for the estimation of maximal vasoconstriction and vasodilatation of mice superior mesenteric artery at 37 °C. A: phenylephrine was used to evoke vasoconstriction with EC50 of 10−6 M. After vessel calibration, the maximal change in luminal diameter evoked by Phe was 9.67%. B: sodium nitroprusside was used to demonstrate the vasodilatation of preconstricted arterial segments; maximal vasodilatation of 3.74% was evoked by 10−4 M sodium nitroprusside compared to the initial diameter. The maximum constriction/vasodilatation was arbitrarily set as 100%. [file 40001_2018_335_MOESM2_ESM.pdf]

Additional file 2

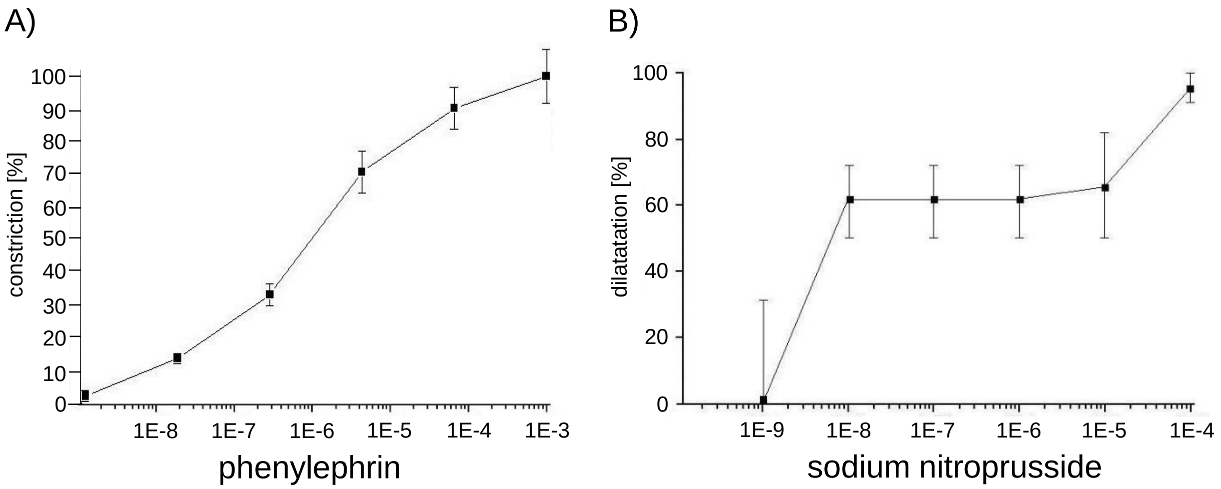

Dose response curves for the estimation of maximal vasoconstriction and vasodilatation of mice superior mesenteric artery at 37°C. A: phenylephrin was used to evoke vasoconstriction with EC_{50} of 10^{-6} M. After vessel calibration the maximal change in luminal diameter evoked by Phe was 9.67%.

B: sodium nitroprusside was used to demonstrate vasodilatation of preconstricted arterial segments, maximal vasodilatation of 3.74% was evoked by 10^{-4} M sodium nitroprusside compared to initial diameter. The maximum constriction / vasodilataion was arbitrarily set as 100%.
